# Prediction of hepatic lymph node metastases based on magnetic resonance imaging before and after preoperative chemotherapy in patients with colorectal liver metastases underwent surgical resection

**DOI:** 10.1186/s40644-023-00529-y

**Published:** 2023-02-21

**Authors:** Hai-bin Zhu, Da Xu, Xue-Feng Sun, Xiao-Ting Li, Xiao-Yan Zhang, Kun Wang, Bao-Cai Xing, Ying-Shi Sun

**Affiliations:** 1 Key Laboratory of Carcinogenesis and Translational Research (Ministry of Education/Beijing), Department of Radiology, Peking University Cancer Hospital & Institute, 52 Fu Cheng Road, Hai Dian District, Beijing, 100142 China; 2 Key Laboratory of Carcinogenesis and Translational Research (Ministry of Education/Beijing), Hepatopancreatobiliary Surgery Department I, Peking University Cancer Hospital & Institute, 52 Fu Cheng Road, Hai Dian District, Beijing, 100142 China

**Keywords:** Colorectal liver metastases, Hepatic lymph node, Magnetic resonance imaging, Diffusion-weighted imaging, Survival

## Abstract

**Background:**

Patients with colorectal liver metastases (CRLM) combined with hepatic lymph node (HLN) metastases have a poor prognosis. In this study, we developed and validated a model using clinical and magnetic resonance imaging (MRI) parameters to predict HLN status before surgery.

**Methods:**

A total of 104 CRLM patients undergoing hepatic lymphonodectomy with pathologically confirmed HLN status after preoperative chemotherapy were enrolled in this study. The patients were further divided into a training group (*n* = 52) and a validation group (*n* = 52). The apparent diffusion coefficient (ADC) values, including ADC_mean_ and ADC_min_ of the largest HLN before and after treatment, were measured. rADC was calculated referring to the target liver metastases, spleen, and psoas major muscle (rADC_-LM_, rADC_-SP,_ rADC_-m_). In addition, ADC change rate (Δ% ADC) was quantitatively calculated. A multivariate logistic regression model for predicting HLN status in CRLM patients was constructed using the training group and further tested in the validation group.

**Results:**

In the training cohort, post-ADC_mean_ (*P* = 0.018) and the short diameter of the largest lymph node after treatment (*P* = 0.001) were independent predictors for metastatic HLN in CRLM patients. The model’s AUC was 0.859 (95% CI, 0.757-0.961) and 0.767 (95% CI 0.634-0.900) in the training and validation cohorts, respectively. Patients with metastatic HLN showed significantly worse overall survival (*p* = 0.035) and recurrence-free survival (*p* = 0.015) than patients with negative HLN.

**Conclusions:**

The developed model using MRI parameters could accurately predict HLN metastases in CRLM patients and could be used to preoperatively assess the HLN status and facilitate surgical treatment decisions in patients with CRLM.

**Supplementary Information:**

The online version contains supplementary material available at 10.1186/s40644-023-00529-y.

## Introduction

More than 50% of patients with colorectal cancer will develop liver metastases [1]. For patients with colorectal liver metastases (CRLM), surgical resection offers the best opportunity to achieve long-term survival, with a 5-year survival rate of 40-50% [2, 3]. However, about 10-31% of CRLM patients concomitantly show hepatic lymph node (HLN) metastases, which is generally considered a contraindication for liver resection [4–6]. With the advancement of systemic therapy and localized treatment in recent years, the indications for resection of CRLM have gradually expanded. Yet, the prognosis of patients with positive HLN is still poor even after complete resection of all hepatic metastases and HLN, with a 5-year survival rate of 10-20%. Therefore, determining the status of HLN is crucial for selecting further treatment strategies [7, 8].

At present, pathology examination of lymph nodes remains the golden standard to assess the status of HLN in CRLM patients. Some studies explored the feasibility of surgeons’ intraoperative palpation of the hepatoduodenal ligament, an important anatomical pathway of the extension of disease, during liver resection, but the sensitivity and specificity of this method are only 67 and 79%, respectively [9]. Therefore, there’s still lack of reliable preoperative methods to assess the status of HLN in patients with CRLM.

Over the years, non-invasive imaging tools have been gradually used to assess lymph node status in patients with cancer. Computer tomography (CT), positron emission tomography (PET), ultrasound, and magnetic resonance imaging (MRI) are some of the most common imaging tools for assessing lymph node status. Yet, diagnostic information provided by CT is mainly based on the enhancement pattern, tumor shape, and diameter, which limits the accuracy when judging tumor activity and function of HLN [7]. Rau et al. [9] found that the short diameter of HLN > 1.5 cm and morphologically full shape detected on CT can be used for HLN judgment, but the sensitivity was only 33%. Similarly, Jaeck et al. [7] failed to predict HLN metastases using preoperative CT in CRLM patients. Therefore, the value of CT in predicting HLN status remains controversial. Fluoro-18-deoxyglucose positron emission tomography (PET) shows high sensitivity for detecting extrahepatic metastases [10, 11]. However, since PET-CT mainly depends on metabolic status rather than size, it is unreliable in discriminating the small lymph nodes with micrometastases or inflammation. Besides, the high cost and lack of repeatability also limited its clinical application [12].

Magnetic resonance imaging (MRI) has been routinely used in patients with CRLM, especially in detecting small liver metastases, with sensitivity as high as 89-96% and specificity of 86-88% [13]. Previous studies explored the use of MRI in diagnosing pelvic lymph node metastases of rectal cancer, with an accuracy rate of about 62-85% [14, 15]. However, the main parameter in these studies was the lymph node size (a cut-off of 8 mm for the pelvis and 10 mm for the abdomen), which limits its diagnostic accuracy. The advantages of MRI is that functional sequences increase their discriminating ability. For example, diffusion-weighted imaging (DWI), in combination with the quantitative calculation of the apparent diffusion coefficient (ADC) value, can be used to show the random Brownian motion of water molecules at the microscopic level in the biological tissues. Due to the dense arrangement of tumor cells and rich fibrous matrix in tumor tissue, the free diffusion of water molecules is restricted, resulting in a high DWI signal and a reduction of ADC value. In contrast, ADC value is significantly increased in non-metastatic and benign tissue. DWI has been more and more applied in determining tumor status and evaluating therapeutic response after treatment [16, 17]. Yet, to date, there is still a lack of studies using functional MRI parameters to determine the status of HLN.

In this study, we explored the value of clinical and functional MRI parameters in assessing the HLN status in patients with CRLM before and after preoperative chemotherapy.

## Materials and methods

### Patients

**Fig. 1:**
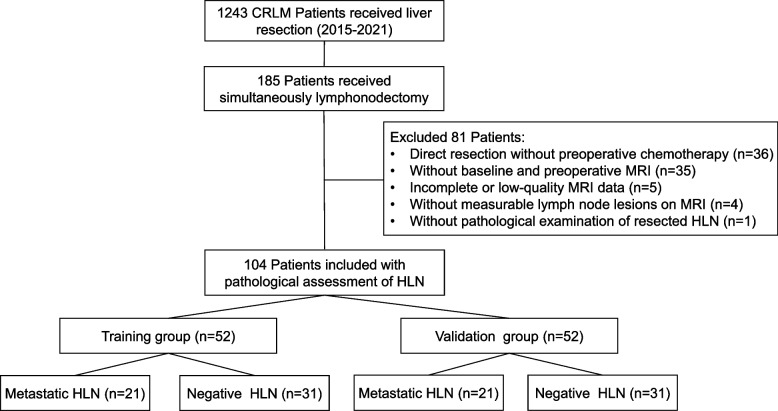
Study flowchart

CRLM patients with suspicious HLN on preoperative MRI who underwent hepatectomy and HLN resection after neoadjuvant chemotherapy in the HPB Surgery Ward I at Peking University Cancer Hospital and Institute (Beijing, China) between January 2012 and June 2021 were selected. The inclusion criteria were: 1) received at least two cycles of neoadjuvant chemotherapy; 2) received MRI examinations before neoadjuvant chemotherapy (baseline point) and after the last cycle of neoadjuvant chemotherapy within 1 month before surgery (preoperative point); 3) MRI examinations including T2WI and DWI sequence with reliable image quality; 4) at least one HLN was present on the MRI image at the baseline with the short diameter of the largest lymph node > 5 mm. Exclusion criteria were the following: 1) received hepatectomy without HLN resection; 2) without pathological examination of resected HLN; 3) no preoperative chemotherapy; 4) without measurable HLN > 5 mm at baseline MR; 5) unclear images. A flow diagram summarizing initial candidates and each exclusion procedure are shown in Fig. 1.

The ethical review board committee approved this study of Peking University Cancer Hospital and Institute (Beijing, China). The informed consent was waived due to its retrospective design.

### MRI examination

Patients were scanned on a 1.5 T MRI device (Signa Excite II; GE Healthcare, Milwaukee, WI, USA) using an 8-channel phased array body coil with the patient in the supine position. Conventional MRI protocols for liver scanning included coronal and axial T2WI, axial fat-suppressed T2WI, DWI, and dynamic contrast-enhanced scan sequences. The corresponding parameters of the axial fast recovery spin echo T2WI sequence (FSE-T2WI) were: repetition time (TR)/echo time (TE) = 12,630/70 ms; matrix = 288 × 224; thickness = 6 mm; interval = 1 mm; FOV =380 × 380 mm^2^. The DWI sequence was a free-breathing, single excitation, echo-planar imaging (EPI) sequence with b values of 0, 20, 50, 100, 200, 600, 800, 1000, 1200, and 1500 s/mm^2^, respectively; the total acquisition time is about 6 minutes and 19 seconds. Imaging parameters of the DWI sequence included TR/TE = 3000/80 ms; matrix size = 128 × 90; thickness = 6 mm; interval = 1 mm; FOV = 380 × 380 mm^2^. Details of the MRI parameters are shown in Table S1.

### Quantitative imaging analysis

**Fig. 2:**
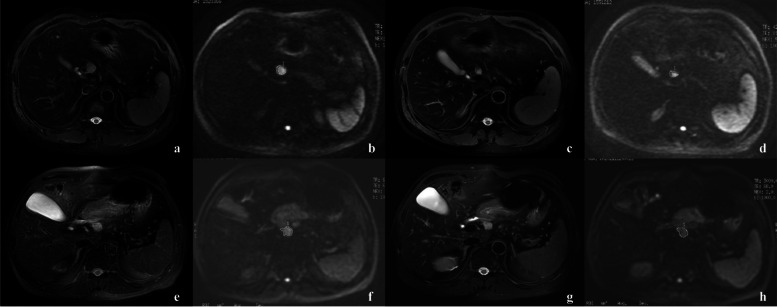
Two examples of MRI images from CRLM patients, including the T2WI and DWI before and after treatment. **a**-**d** A short diameter of HLN shrinkage from 16 mm (**a**) to 11 mm (**c**). The ADC value calculated from the DWI sequence (**b**, **d**) slightly increased from 1140 to 1230 mm^2^/s. The lymph node was pathologically confirmed to be a metastatic lymph node. **e**-**h** A short diameter of HLN shrinkage from 15 mm (**e**) to 12 mm (**g**) after treatment. The ADC value calculated from the DWI sequence (**f**, **h**) increased from 1330 to 1650 mm^2^/s. The lymph node was pathologically confirmed to be a non-metastatic lymph node

All MRI data were further evaluated at a specific imaging workstation (ADW 4.4; GE Healthcare). MR images were evaluated by two radiologists (H.B.Z and X.Y.Z). The two radiologists adopted a consensus evaluation method, performed one-to-one correspondence between surgically removed HLN and imaging localization according to the surgical records, and selected the largest HLN as the target lesion. ADC values were calculated using a single exponential model with b value = 0 and 1000 s/mm^2^. The region of interest (ROI) was placed on the DWI image by free delineation, DCE-MRI and T2WI images were used as references. The corresponding ADC map was generated by using the built-in software, including ADC_mean_ and ADC_min_ values before and after treatment (Fig. 2). At the same time, ADC values of the largest liver metastases, the spleen (at the largest diameter of the spleen, avoiding the major vessel branch, ROI of 100-200 mm^2^), and the bilateral psoas muscle (at the spleen hilar slice, ROI of 100-200 mm^2^) were measured. The rADC was calculated as follows: rADC_-LM_ = ADC_mean_ of HLN/ADC_mean_ of target liver metastases; rADC_-sp_ = ADC_mean_ of HLN/ADC of the spleen; rADC_-m_ = ADC_mean_ of HLN/average of ADC of the bilateral psoas muscle. In addition, the change of ADC value before and after treatment was quantitatively calculated, Δ% ADC = (post-ADC - pre-ADC)/ pre-ADC.

### Perioperative management and definitions

Multidisciplinary team meetings were scheduled on a weekly basis in our center for patients with CRLM. The clinical information of the patients was collected, including age, gender, location of the primary tumor (left-side or right-side), pathological T and N stages of the primary tumor, synchronous or metachronous liver metastases, and the number of tumors (single or multiple), RAS, BRAF gene status (mutant or wild type) and microsatellite instability (MSI) status, serum carcinoembryonic antigen (CEA), and CA19-9 tumor marker levels before and after treatment. Preoperative chemotherapy included irinotecan- or oxaliplatin-containing combination regimens with or without targeted agents (bevacizumab and cetuximab). The largest two liver metastases were selected as target lesions, and T2WI images before and after chemotherapy were calculated to measure the change rate of the long-axis of the tumor according to the RECIST1.1 standard. Complete response (CR) and partial response (PR) were classified as the response group, while stable disease (SD) and progressive disease (PD) were classified as the non-response group.

### Surgical technique

Resectable liver metastases were defined as the complete removal of macroscopic tumors, with at least a 30% future liver remnant (FLR) or a remnant liver-to-body weight ratio > 0.7, with sufficient blood inflow and outflow of the liver. Intra-operative ultrasound or contrast-enhanced ultrasound was routinely performed during the hepatectomy to detect the presence of undetected lesions. For patients with multiple liver metastases, surgical resection in combination with local ablative treatments was used to achieve a status of no evidence of disease and preserve liver parenchyma to the greatest extent.

The HLN was defined as previously described: nodes located along the hepatoduodenal ligament (along the proper hepatic artery, portal vein, bile duct, and retro-pancreatic head); along the common hepatic artery and coeliac artery (along the coeliac, common hepatic, and left gastric arteries) [8]. Because standard regional hilar lymph node dissection was not routinely performed, suspected HLN were resected based on preoperative imaging location and described in the surgical record. All hematoxylin and eosin (H&E) stained slides of the HLN resection block were reviewed and compared with the resected lymph node area according to surgical records and imaging localization.

### Follow up

Routine examinations were tested every 3-6 months. Overall survival (OS) was measured from the operation date until the date of death or last follow-up. Recurrence-free survival (RFS) was measured from the operation date to local recurrence, distant metastasis, or last follow-up.

### Statistical analysis

Continuous and categorical variables are described as mean ± quasi-deviation and numbers with percentages, respectively. Independent-samples t/Mann–Whitney or chi-square tests were used to compare characteristics between the MHLN (metastatic HLN) and NHLN (non-metastatic HLN) groups. Multivariable logistic regression using a forward stepwise approach was conducted to test independent factors associated with hilar node status, and a predictive model was constructed accordingly. The receiver operating characteristic (ROC) curve was used to evaluate the diagnostic performance, and the area under the ROC curve (AUC) with its 95% confidence interval was calculated. The cutoffs were determined using the maximum Youden’s method, and sensitivity, specificity, positive predictive value (PPV), and negative predictive value (NPV) were calculated. Kaplan-Meier curves with log-rank estimates were used to compare the MHLN and NHLN survival outcomes. *P* value < 0.05 indicated statistical significance. SPSS 25.0 (IBM Corporation, Armonk, NY, USA) was used for all statistical analyses.

## Results

A total of 1243 CRLM patients underwent surgical resection at our center from January 2015 to June 2021, of whom 104 CRLM patients underwent lymphonodectomy and were pathology-confirmed with HLN status after preoperative chemotherapy; these 104 were enrolled in this study.

### MRI parameters associated with MHLN

The patients were further divided into two groups according to the order of imaging diagnosis time: the training group (*n* = 52) and the validation group (*n* = 52). Both groups included 21 patients with MHLN and 31 patients with NHLN. The patient characteristics are summarized in Table 1. The clinical and imaging characteristics of the patients were not significantly different except for the number of CRLM (*P* = 0.027) and post-CEA level (*P* = 0.049).

**Table 1:** Clinical and MRI features of patients in the training and validation group

		Training group	Validation group	*P* value
HLN	NHLN	31	31	/
	MHLN	21	21	
Gender	male/female	12/40	19/33	0.133
Age		52.85 ± 9.19	56.69 ± 10.86	0.054
BMI		24.37 ± 3.04	23.71 ± 3.11	0.273
Primary location	Right−/left-side	11/41	11/41	/
Differentiation	Low to moderate/High	51/1	51/1	/
T stage of primary tumor	T1 + 2/T3 + 4	5/47	2/50	0.437
N stage of primary tumor	N0/N+	4/48	10/42	0.149
Gene	RAS-wild	32	32	0.650
	RAS-mutation	19	17	
	Braf-mutation	1	3	
Simultaneous liver metastases	No/Yes	13/39	10/42	0.478
Distribution	Solitary/Bilateral	15/37	23/29	0.103
Number of CRLM	≤5/>5	24/28	38/14	0.027*
Size (mm)		43.17 ± 28.90	37.46 ± 20.87	0.420
RECIST evaluation	Response/Non- Response	31/21	28/24	0.553
pre-CEA	≤5/>5 ng/ml	8/37	12/37	0.368
pre-CA199	≤40/>40 U/ml	16/31	17/30	0.829
post-CEA	≤5/>5 ng/ml	19/33	29/23	0.049*
post-CA199	≤40/>40 U/ml	29/29	29/19	/
Shape of HLN	Regular/Irregular	43/9	40/12	0.464
Signal intensity of HLN	Homogeneous/Hetergeneous	41/11	37/15	0.365
Short axis of HLN at baseline	mm	8.54 ± 3.63	8.63 ± 4.44	0.904
Long axis of HLN at baseline	mm	16.17 ± 6.77	15.17 ± 6.48	0.443
Pre-ADC_mean_	mm^2^/s	1513.08 ± 311.09	1590.10 ± 333.12	0.226
Pre-ADC_min_	mm^2^/s	1107.94 ± 300.83	1169.13 ± 341.12	0.334
Pre-rADC_-lm_		1.21 ± 0.29	1.38 ± 0.32	0.006
Pre-rADC_-sp_		1.82 ± 0.34	1.90 ± 0.44	0.330
Pre-rADC_-m_		1.11 ± 0.24	1.18 ± 0.24	0.182
Short axis of HLN at endpoint	mm	8.15 ± 3.10	7.81 ± 3.30	0.583
Long axis of HLN at endpoint	mm	15.58 ± 6.59	14.42 ± 6.32	0.364
Post-ADC_mean_	mm^2^/s	1563.46 ± 272.00	1575.88 ± 374.27	0.847
Post-ADC_min_	mm^2^/s	1166.77 ± 289.29	1156.79 ± 360.96	0.977
Post -rADC_-lm_		1.25 ± 0.32	1.26 ± 0.38	0.869
Post -rADC_-sp_		1.93 ± 0.45	1.86 ± 0.40	0.388
Post-rADC_-m_		1.11 ± 0.22	1.16 ± 0.28	0.380
ΔADC		6.04 ± 24.63	3.39 ± 25.67	0.435

*ADC* Apparent diffusion coefficient, *CEA* Carcinoembryonic antigen, *CA19-9* Carbohydrate antigen 19-9, *CRLM* Colorectal liver metastases, *HLN* Hepatic lymph node, *MHLN* Metastatic hepatic lymph node, *NHLN* Non-metastatic hepatic lymph node, *RECIST* Response Evaluation Criteria In Solid Tumors

*P values that are significantly different between training group and validation group

In the training cohort, the signal intensity of HLN at baseline (*P* = 0.019), the short diameter of lymph nodes before and after treatment (*P* = 0.030, *P* < 0.001), the long diameter of lymph nodes after treatment (*P* = 0.045), post-ADC_mean_ (*P* = 0.004), and post-rADC_-m_ (*P* = 0.021) were different between MHLN and NHLN groups (Table 2).

**Table 2:** Univariate and multivariate analysis of clinical and MRI factors for prediction of hilar lymph nodes in the training group

		Univariate analysis			Multivariate Analysis	
		MHLN (*n* = 31)	NHLN (*n* = 21)	*P* value	OR (95% confidence interval)	*P* value
Gender	male/female	8/23	4/17	0.741		
Age		52.90 ± 9.71	52.76 ± 8.58	0.141		
BMI		24.51 ± 3.21	24.16 ± 2.86	0.358		
Primary location	Right−/left-side	7/24	4/17	> 0.999		
Differentiation	Low to moderate/High	30/1	20/1	> 0.999		
T stage of primary tuomor	T1 + 2/T3 + 4	1/30	4/17	0.145		
N stage of primary tuomor	N0/N+	3/28	1/20	0.639		
Gene	RAS-wild	20	12	0.540		
	RAS-mutation	11	8			
	Braf-mutation	0	1			
Simultaneous liver metastases	No/Yes	6/25	7/14	0.253		
Distribution	Solitary/Bilateral	8/23	7/14	0.557		
Number of CRLM	≤5/>5	10/21	13/8	0.277		
Size (mm)		43.00 ± 30.89	43.43 ± 26.44	0.958		
RECIST evaluation	Response/Non- Response	21/10	10/11	0.147		
pre-CEA	5 ng/ml	4/24	4/15	0.697		
pre-CA199	U/ml	11/17	5/14	0.357		
post-CEA	5 ng/ml	12/19	7/14	0.693		
post-CA199	U/ml	18/11	11/8	0.772		
Shape of HLN	Regular/Irregular	26/5	17/4	> 0.999		
Signal intensity of HLN	Homogeneous/Hetergeneous	28/3	13/8	0.019*		
Short axis of HLN at baseline	mm	7.65 ± 2.79	9.86 ± 4.35	0.030*		
Long axis of HLN at baseline	mm	15.52 ± 6.74	17.14 ± 6.86	0.401		
Pre-ADC_mean_	mm^2^/s	1528.06 ± 313.46	1490.95 ± 313.89	0.678		
Pre-ADC_min_	mm^2^/s	1115.45 ± 281.98	1096.86 ± 333.60	0.829		
Pre-rADC-_lm_		1.24 ± 0.32	1.17 ± 0.25	0.411		
Pre-rADC-_sp_		1.85 ± 0.37	1.79 ± 0.31	0.553		
Pre-rADC_-m_		1.13 ± 0.23	1.09 ± 0.26	0.598		
Short axis of HLN at endpoint	mm	6.77 ± 2.17	10.19 ± 3.19	< 0.001*	1.687(1.226-2.322)	0.001*
Long axis of HLN at endpoint	mm	14.06 ± 6.32	17.81 ± 6.48	0.045*		
Post-ADC_mean_	mm^2^/s	1649.90 ± 260.52	1435.86 ± 241.20	0.004*	0.996(0.994-0.999)	0.018*
Post-ADC_min_	mm^2^/s	1228.77 ± 289.57	1075.24 ± 269.96	0.060		
Post -rADC-_lm_		1.29 ± 0.34	1.18 ± 0.28	0.239		
Post -rADC-_sp_		2.01 ± 0.40	1.81 ± 0.50	0.121		
Post-rADC_-m_		1.17 ± 0.24	1.03 ± 0.18	0.021*		
ΔADC		9.65 ± 22.55	0.72 ± 27.10	0.203		

*P values that are significantly different between MHLN group and NHLN group

In the multivariate logistic regression analysis, post-ADC_mean_ (OR, 0.996; 95% confidence interval, 0.994-0.999, *P* = 0.018) and short diameter of the largest lymph node after treatment (OR = 1.687; 95% confidence interval, 1.226-2.32, *P* = 0.001) were found to be independent predictors for MHLN in CRLM patients. The results are shown in Table 2.

### Construction of MRI model for predicting MHLN and model validation

**Fig. 3:**
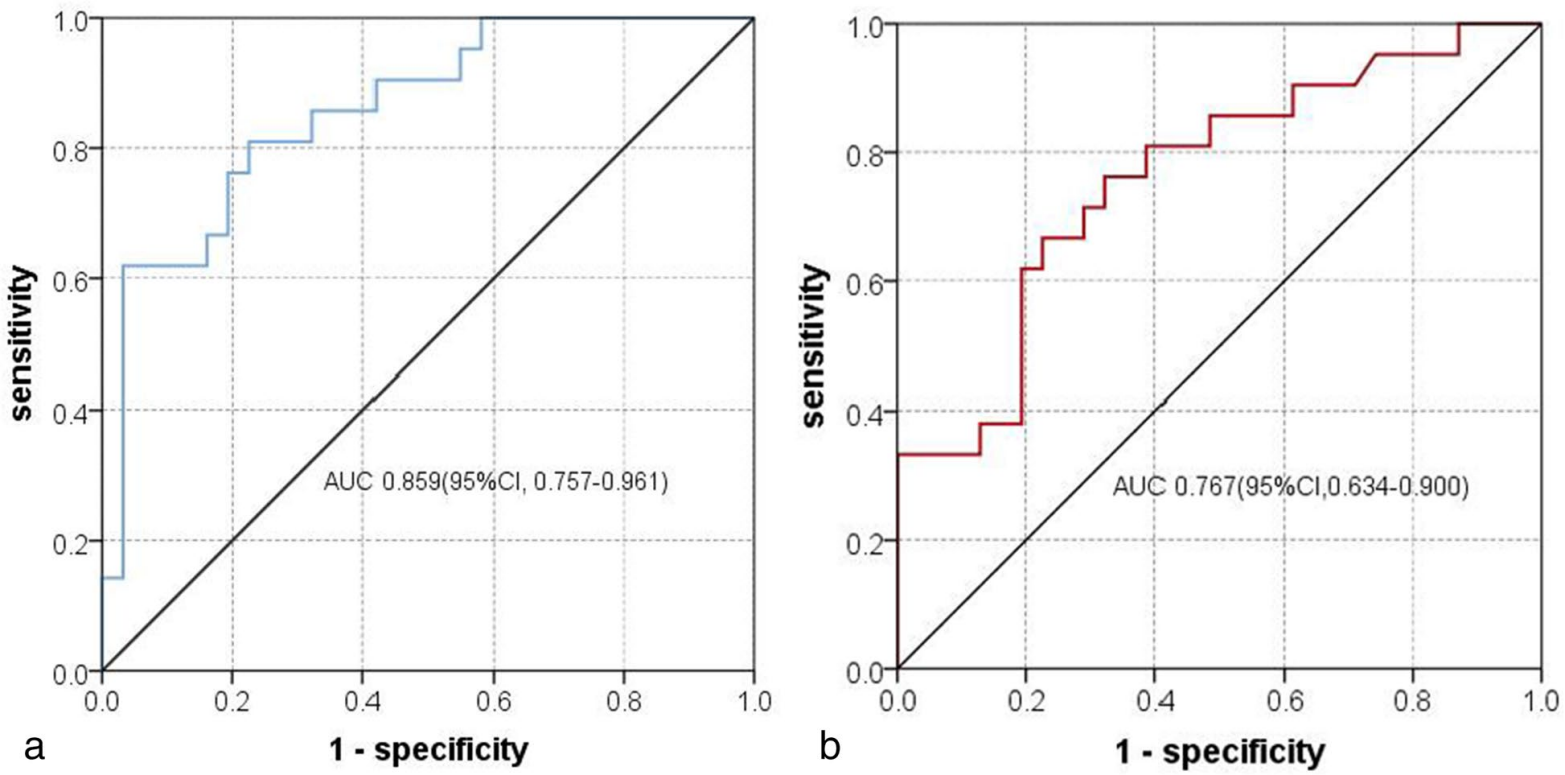
Receiver operating characteristics curves of functional MRI model predicting MHLN in the training cohort (**a**) and validation cohort (**b**)

The above independent predictors of MHLN in the multivariate logistic regression analysis were integrated into a functional MRI model predicting MHLN. In the training cohort, the model’s AUC was 0.859 (95% CI, 0.757-0.961), with sensitivity, specificity, positive predictive value, negative predictive value, and accuracy of 76.2, 77.4, 69.6, 82.8, and 76.9%, respectively (Fig. 3a). In the validation cohort, the AUC of the model was 0.767 (95% CI 0.634-0.900), with sensitivity, specificity, positive predictive value, negative predictive value, and accuracy of 52.4, 80.6, 64.7, 71.4, and 69.2%, respectively (Fig. 3b, Table 3).

**Table 3:** Diagnostic performance of model in predicting HLN status in CRLM patients in the training and validation cohort

	AUC	Cut off value	sensitivity	Specificity	Positive predictive value	Negative predictive value	Accuracy
Training cohort	0.859(0.757-0.961)	38%	76.2	77.4	69.6	82.8	76.9
Validation cohort	0.767(0.634-0.900)	38%	52.4	80.6	64.7	71.4	69.2

*AUC* Area Under Curve

### Survival analysis

**Fig. 4:**
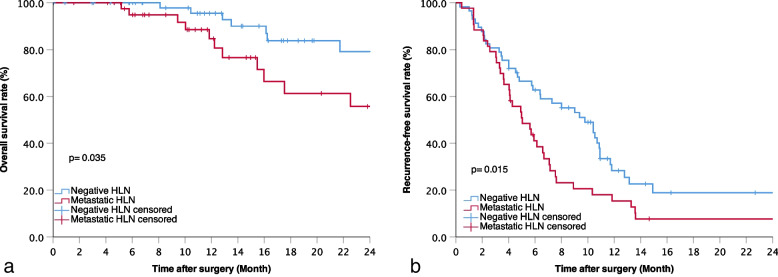
Kaplan-Meier analyses of OS (**a**) and RFS (**b**) for patients with MHLN and NHLN (OS, *P* = 0·035; RFS, *P* = 0.015)

Of the 104 patients who underwent lymphonodectomy, 42 (40.4%) were pathologically confirmed with MHLN and 62 (59.6%) with NHLN. After a median follow-up time of 14 months, patients with MHLN showed worse OS (Fig. 4a) and RFS (Fig. 4b) than patients with NHLN (2-year OS: 79.1% versus 55.7%, *P* = 0.035; 2-year RFS: 18.9% versus 7.7%, *P* = 0.015).

## Discussion

HLN metastases are important factors for poor prognosis in patients with CRLM, even after resection. In this study, we found that the survival of patients with MHLN was significantly worse than those with NHLN. Patients with heavy metastases burden combined with HLN metastases have poor biological behavior and are generally considered a contraindication for liver resection, while for those with negative HLN or metastatic HLN responding well to neoadjuvant chemotherapy, a more aggressive resection strategy can be considered to prolong survival. Thus, preoperative prediction of HLN status is of vital importance in guiding clinical decisions. Unfortunately, there is still a lack of well-accepted standards in the preoperative prediction of HLN status. To the best of our knowledge, this is the first study that reported on the relationship between MRI features and lymph node status in CRLM patients who underwent HLN dissection, showing that the ADC value of lymph nodes (post-ADC_mean_) and the short diameter of the largest lymph node after treatment were independent predictors for prediction of the status of HLN; a high AUCs was obtained for both training (0.859, 95% CI: 0.757-0.961) and validation cohorts (0.767, 95% CI: 0.634-0.900), respectively.

MRI is the most commonly used method for evaluating tumor aggressiveness and treatment response in CRLM patients. The advantage of MRI lies in multi-sequence imaging, which can provide more functional information in comparison with CT. As an effective non-invasive method, DWI has been widely used in evaluating metastatic lymph nodes in rectal, cervical, and nasopharyngeal cancer [18–20]. This study further confirmed that the post-ADC_mean_ of MHLN was lower than that of NHLN, which was consistent with previous studies [21, 22]. The decreased ADC value of MHLN may be attributed to a higher tumor cell density and fibrosis in the extracellular space caused by collagen deposition, narrowing of the intracellular and extracellular spaces, and limiting the free movement of water molecules. In contrast, negative lymph nodes or tumor cells that disappear after treatment are characterized by a loss of tumor structural integrity and reduced cell density, resulting in increased cell membrane permeability and acceleration diffusion of water molecules [23–25]. We observed that the ADC value of HLN after chemotherapy was elevated after treatment, which further supported this speculation. Therefore, the post-ADC_mean_ can effectively discriminate between negative and metastatic HLN.

Our data suggested that ADC values at baseline before treatment could not predict the status of HLN in patients with CRLM after neoadjuvant therapy. Previous studies also explored the value of baseline ADC in predicting the chemotherapy response of liver metastases, although these research conclusions were incongruent. Matsushima et al. [26] used DWI to predict the chemotherapy response of CRLM patients receiving bevacizumab-containing chemotherapy and found no significant difference in ADC values between the chemo response group (CR + PR) and non-response group (SD + PD). In contrast, Drewes et al. [27] found that pretreatment mean ADC in the responder group was significantly lower in comparison with the non-responder group, with the best cut-off threshold of 1.2 × 10^− 3^ mm^2^/s. The following reasons might contribute to the failure of baseline ADC to predict HLN status: (1) although the ADC value measured by the whole volume can better reflect the heterogeneity of the tumor, the necrotic area indicating hypoxia was included, which would further induce a decrease in ADC. Therefore, the overlap of pre-ADC_mean_ between MHLN and NHLN was increased. (2) The micrometastases of the lymph node are sometimes cleared after systematic chemotherapy, resulting in an inaccurate prediction of lymph node status by initial ADC values; (3) the degree of tumor differentiation would also influence the baseline ADC values.

A previous study found that preoperative rADC_-LM_ is helpful in identifying HLN status. The optimal cut-off point of rADC was 1.15, with corresponding AUC, sensitivity, and specificity of 0.80, 0.69, and 0.93, respectively [18]. However, none of the rADC values (pre- or post-treatment) showed efficacy in identifying MHLN in this study. These results might be explained by the fact that the previous study included patients who underwent direct resection without receiving adjuvant chemotherapy (34.4%, 11/32), while all patients included in this study received preoperative chemotherapy. Chemotherapy could shrink the tumor and increase necrosis within the tumor, which further leads to a decrease in the predictive value of rADC. Besides, the ROI segmentation algorithm used in the study could also affect the results. In the present study, the ROI was drawn along the border of the tumor as large as possible at the largest slice of the target tumor, avoiding adjacent liver parenchyma. In contrast, the previous study only outlined the solid component of the target tumor. To minimize the impact of the ROI method on the results, we additionally used the spleen and psoas muscle as reference points. Interestingly, all these rADC values were not statistically different.

This study found that the short diameter of the largest lymph node was an independent predictor for evaluating the status of the HLN. Hida et al. [14] analyzed the lateral lymph node status of rectal cancer on MR images in 322 patients, finding short diameter ≥ 5 mm could well differentiate metastatic and negative lymph nodes, with a significant difference in survival between the two groups. Moreover, Shi et al. [28] explored the clinicopathological and radiological features of 127 resected pancreatic ductal adenocarcinoma and determined that the short diameter of the largest lymph node was an independent predictor for lymph node status (OR, 1.528; *P* = 0.011). It should be noted that many other parameters, such as short/ long diameter ratio, and lymph node number, are also considered to reflect metastatic burden and prognosis in previous studies [29, 30]. However, these factors were not significant in predicting the status of HLN in this study.

In addition to imaging examinations, some studies also explored the clinical characteristics in predicting high-risk CRLM patients accompanied by MHLN. Oussoultzoglou et al. [31] found multiple hepatic metastases (three or more), nodules located on segments 4 and 5, and elevated CEA (over 20 μg/dl) were at risk for MHLN. Another study found that primary tumor lymph node metastases and intrahepatic recurrence requiring repeat hepatectomy were significant risk factors for MHLN on multivariable analysis [8]. In our study, the number of liver metastases was also associated with MHLN in univariate analysis. Unfortunately, this factor was not significant in multivariate analysis. We should note that different study cohorts and the bias in patient recruitment might also affect the clinical predictive factors for MHLN. Considering the above results, we suspected that MHLN was more commonly found in patients with heavy tumor burden. However, these clinical parameters can only be regarded as increased risk factors for MHLN but cannot be used to assess the status of HLN objectively.

There are several limitations in the present study. First, the positive rate of MHLN reported in previous studies was about 10-31% in CRLM patients who underwent lymphonodectomy [5, 7, 8, 32–34]. The positive rate in this study was 40.4%, which might be explained by the fact that all the patients in the study received neoadjuvant chemotherapy and MRI examinations, and the status of the HLN has been strictly screened before lymphonodectomy, leading to a relatively high positive rate. Second, there may be bias in measuring ADC values by different observers, especially when the lymph nodes are small, which is also a common problem in measuring ADC values. The lack of repeated measurement is one of the shortcomings of this study. Thirdly, the largest lymph node was selected as the target lesion for imaging and pathological examinations. Therefore, some small lymph nodes might be ignored, which might not fully represent the overall status of all HLN. In addition, as a retrospective study, a relatively small sample size and bias in patient selection were inevitable. Traditionally, 70-80% of the patients are usually used to construct the model in the training cohort for ensuring the stability of model. Considering the total patients included in this study were only 104 cases, if 70-80% of the patients are used to construct the model, too small sample size (about 20 cases) in the validation group may also affect the stability of results in the validation group. To ensure the stability and accuracy of model, we split patients into two groups symmetrically according to imaging time. Thus, the results of the study are inclined to “formulate a research hypothesis”, much larger samples from multicenter were needed to validate the advantage and reproducibility of this model.

## Conclusions

A model combining post-ADC_mean_ value and short diameter of the largest lymph node after treatment can be used to predict lymph node status in CRLM patients receiving neoadjuvant therapy preoperatively, which can facilitate the personalized treatment of CRLM patients with HLN.

## Supplementary Information

**Additional file 1: Table S1.** MRI Parameters.

## Data Availability

Data can be obtained with appropriate reason by request to the corresponding author through email sys27@163.com.

## Abbreviations

ADC: Apparent diffusion coefficient
AUC: Area under the curve
CRLM: Colorectal liver metastases
CT: Computed tomography
DWI: Diffusion weighted imaging
EPI: Echo-planar imaging
FLR: Future liver remnant
FSE: Fast spin echo
FOV: Field of view
HLN: Hepatic lymph node
MHLN: Metastatic hepatic lymph node
MRI: Magnetic resonance imaging
MSI: Microsatellite instability
NHLN: Non-metastatic hepatic lymph node
OS: Overall survival
PET: Positron emission tomography
RFS: Recurrence-free survival
ROC: Receiver operating characteristic
TE: Echo time
TR: Repetition time
