# Supplementary material for: Prediction of hepatic lymph node metastases based on magnetic resonance imaging before and after preoperative chemotherapy in patients with colorectal liver metastases underwent surgical resection
Source: Cancer Imaging. 2023 Feb 21;23:18. doi: 10.1186/s40644-023-00529-y (PMC9942330; doi:10.1186/s40644-023-00529-y)
Supplement: Supplementary file 1 — Additional file 1: Table S1. MRI Parameters. [file 40644_2023_529_MOESM1_ESM.docx]

**Table S1.** MRI Parameters

| Parameters | T1-weighted imaging | T2-weighted imaging | DWI imaging | DCE imaging |
| --- | --- | --- | --- | --- |
| Sequences | Axial FSE T1WI | Axial FSE T2WI | Axial SE-EPI | Axial LAVA flex |
| Repetition time/Echo time (msec) | 6/min | 12,630/70 | 3000/80 | Min/min |
| Flip angle (degrees) | 15 | 110 | 90 | 15 |
| Field of view (mm^2^) | 380 x 380 | 380 x 380 | 380 x 380 | 380 x 380 |
| Matrix (frequency × phase) | 224 x 192 | 228 x 224 | 128 x 90 | 224 x 192 |
| Slice thickness (mm) | 6 | 6 | 6 | 5 |
| Slice gap (mm) | 1 | 1 | 1 | 0 |

FSE = fast spin-echo; SE = spin-echo; EPI = echo-planar imaging; LAVA = liver acquisition with volume acceleration.
